# *AtDRO1* is nuclear localized in root tips under native conditions and impacts auxin localization

**DOI:** 10.1007/s11103-020-00984-2

**Published:** 2020-03-04

**Authors:** Jessica M. Waite, Tamara D. Collum, Chris Dardick

**Affiliations:** 1 Washington State University Tree Fruit Research and Extension Center, Wenatchee, WA 98801 USA; 2 USDA-ARS Appalachian Fruit Research Station, Kearneysville, WV 25430 USA

**Keywords:** Root system architecture, IGT family, LAZY family, Gravitropic set point, Protein localization

## Abstract

**Abstract:**

*DEEPER ROOTING 1 (DRO1)* contributes to the downward gravitropic growth trajectory of roots upstream of lateral auxin transport in monocots and dicots. Loss of *DRO1* function leads to horizontally oriented lateral roots and altered gravitropic set point angle, while loss of all three *DRO* family members results in upward, vertical root growth. Here, we attempt to dissect the roles of *AtDRO1* by analyzing expression, protein localization, auxin gradient formation, and auxin responsiveness in the *atdro1* mutant. Current evidence suggests AtDRO1 is predominantly a membrane-localized protein. Here we show that VENUS-tagged AtDRO1 driven by the native *AtDRO1* promoter complemented an *atdro1* Arabidopsis mutant and the protein was localized in root tips and detectable in nuclei. *atdro1* primary and lateral roots showed impairment in establishing an auxin gradient upon gravistimulation as visualized with DII-VENUS, a sensor for auxin signaling and proxy for relative auxin distribution. Additionally, PIN3 domain localization was not significantly altered upon gravistimulation in *atdro1* primary and lateral roots. RNA-sequencing revealed differential expression of known root development-related genes in *atdro1* mutants. *atdro1* lateral roots were able to respond to exogenous auxin and *AtDRO1* gene expression levels in root tips were unaffected by the addition of auxin. Collectively, the data suggest that nuclear localization may be important for AtDRO1 function and suggests a more nuanced role for DRO1 in regulating auxin-mediated changes in lateral branch angle.

**Key message:**

DEEPER ROOTING 1 (DRO1) when expressed from its native promoter is predominately localized in Arabidopsis root tips, detectable in nuclei, and impacts auxin gradient formation.

**Electronic supplementary material:**

The online version of this article (10.1007/s11103-020-00984-2) contains supplementary material, which is available to authorized users.

## Introduction

The spatial distribution, or architecture, of a root system has major impacts on plant performance, including anchorage in the soil, access to water and nutrients, and interactions with soil biota. Root system architecture is quantified by numerous parameters. One important architectural parameter is the angle or orientation of root growth. Root orientation can determine the overall width and depth of the root system, which in turn influences the soil layers a plant can grow into, (Kramer 1983; Lynch 2013; Roychoudhry and Kepinski 2015) and is thus a target of interest for crop improvement.

The gene *DEEPER ROOTING 1 (DRO1)* was originally identified in rice from a quantitative trait locus associated with root orientation and overall root system depth (Uga et al. 2013). Rice plants with an intact version of *DRO1* grew deeper roots and performed better in water-limited settings. Since then, a number of studies have characterized *AtDRO1* (*At1g72490*, referred to as *AtDRO1* (Guseman et al. 2017), *LAZY4* (Yoshihara and Spalding 2017), *LZY3* (Taniguchi et al. 2017) or *NGR2* (Ge and Chen 2016)) and two other *DRO* genes in Arabidopsis, and placed them within the larger *IGT* or *LAZY* gene family, with *LAZY* and *TILLER ANGLE CONTROL* genes (Table S1, Yoshihara et al. 2013; Hollender and Dardick 2015; Taniguchi et al. 2017; Yoshihara and Spalding 2017; Guseman et al. 2017; Ge and Chen 2019). Triple mutants of all three *DRO* genes exhibit roots that reverse their gravitropic growth and grow upward against the gravity vector (Ge and Chen 2016; Taniguchi et al. 2017; Yoshihara and Spalding 2017). These results suggest that *DRO* family members additively contribute to setting both lateral and primary root orientation. Intriguingly, they also demonstrate that in the absence of *DRO* genes, the gravitropic set point for root growth is completely reversed – demonstrating the critical role they collectively play in setting overall root architecture. In addition, *DRO* genes have also been shown to additively contribute to shoot gravitropic set point angles along with other *IGT* genes, including *LAZY1* (Taniguchi et al. 2017; Yoshihara and Spalding 2017). Loss of *LAZY* genes in the shoot leads to an inverse gravitropic set point from what was observed upon loss of *DRO1* in the root, i.e. downward growth (Dardick et al. 2013; Uga et al. 2013; Ge and Chen 2016; Taniguchi et al. 2017; Yoshihara and Spalding 2017; Guseman et al. 2017).

The *AtDRO1* promoter has been shown to drive expression of GFP, VENUS, and GUS reporter proteins in root tips, columella cells, and more distally in primary roots and older lateral roots (Yoshihara and Spalding 2017; Guseman et al. 2017; Ge and Chen 2019). The AtDRO1 protein contains all 5 domains conserved among the IGT gene family (Dardick et al. 2013; Yoshihara et al. 2013; Yoshihara and Spalding 2019). The only recognizable motif among these includes an ethylene-responsive element binding factor associated amphiphilic repression-like motif, or EAR-like motif, associated with transcriptional repression (Kagale and Rozwadowski 2010). This motif has been shown in wheat DRO1 to facilitate interaction with the TOPLESS protein at the plasma membrane and nucleus (Ashraf et al. 2019). Other conserved domains have been characterized within the similar LAZY1 protein to be important for nuclear localization, plasma membrane localization, and association with microtubules using transient assays in protoplasts, and Arabidopsis and *N. benthamiana* leaves (Yoshihara et al. 2013; Sasaki and Yamamoto 2015; Yoshihara and Spalding 2019). AtDRO1 protein driven by an estradiol-inducible promoter, was found to localize to the membranes of root epidermal and lateral root cap cells (Ge and Chen 2019). While a nuclear localization signal was predicted, nuclear localization was found to not be required for LAZY1 function in shoot gravitropism (Yoshihara et al. 2013). In contrast, studies in rice found that nuclear localization of LAZY1 was required for functional rescue of shoot gravitropism phenotypes (Li et al. 2019).

Auxin plays a critical role in gravity response. It has been shown that treatment with auxin narrows lateral root angles (Rosquete et al. 2013). Recent work with *DRO* and *LAZY* genes found that corresponding triple and quadruple mutants exhibit impaired lateral auxin transport in response to changes in gravity, as demonstrated through experiments with DR5-VENUS, DII-VENUS, and PIN3-GFP reporter lines (Taniguchi et al. 2017; Yoshihara and Spalding 2017; Ge and Chen 2019). These results support earlier work with *LAZY1* in rice and maize which directly measured differences in auxin levels on upper and lower sides of gravistimulated coleoptiles (Li et al. 2007; Yoshihara and Iino 2007; Dong et al. 2013). In triple mutants lacking either all three *DRO* genes, lacking *LAZY1* and two *DRO* genes, or lacking *LAZY1* and all three *DRO* genes, these reporter signals reverse along with the direction of primary and lateral root growth, although not to the same magnitude as the normal wild-type (WT) response to gravity (Ge and Chen 2016, 2019; Taniguchi et al. 2017; Yoshihara and Spalding 2017). Currently, it is unclear how *IGT* genes mediate these changes. A microarray study in rice showed very few changes in auxin-related gene expression between plants expressing high and low levels of *OsDRO1* (Uga et al. 2013). Polar auxin transport and PINs are required for the phenotypes of *IGT* multiple loss of function mutants (Yoshihara and Spalding 2017; Ge and Chen 2019). Recent work shows that RLD (or BRXL) proteins, which contain a BRX domain, interact with the C-terminal domain of LAZY and DRO proteins and promote translocation from the cytoplasm to the plasma membrane where they mediate PIN3 localization and modulate auxin flow (Furutani et al. 2020).

To date, visualization in live tissues of IGT proteins expressed under their native promoters has not been reported. Here, we attempted to visualize AtDRO1 protein when expressed from its native promoter. Both N- and C-terminal fused VENUS proteins complemented the *atdro1* mutant, however, the C-terminal fusion consistently yielded lateral root tip angles more similar to WT. Under these native conditions, AtDRO1 was difficult to visualize but predominantly found to be expressed in the nuclei of root tip cells. We found reduced auxin asymmetry measured by DII-VENUS signal in *atdro1* mutant plants upon root gravistimulation in primary and lateral roots, and impairment in PIN3-GFP localization. While auxin treatment led to more downward root angles, a similar change occurred in *atdro1* mutants and WT plants and did not alter *AtDRO1* expression. Further, we identified 87 differentially expressed genes (DEGs) in root tips of *atdro1* mutants, which include a number of auxin and root development-related genes. Collectively, the data reveal a potential role for nuclear localization of AtDRO1 and highlight similar, yet potentially complex functions among IGT genes.

## Results

### Expression of VENUS tagged AtDRO1 complements *atdro1* lateral root branch phenotype

*AtDRO1* has previously been shown to be expressed predominantly in roots where it plays a role in determining lateral root branch angles (Taniguchi et al. 2017; Yoshihara and Spalding 2017; Guseman et al. 2017). Previous studies have shown AtDRO1 to be a plasma membrane (PM)-localized protein using protoplasts, transient expression in *N. benthamiana* leaves, or heat-shock induced expression assays (Uga et al. 2013; Taniguchi et al. 2017; Ge and Chen 2019). More recently, AtDRO1-mCherry driven by the native promoter was not able to be visualized in live tissue, but could be seen in the plasma membrane of columella cells in tissue that had been cleared and fixed (Furutani et al. 2020), however the the localization of AtDRO1 protein when expressed from its native promoter in live tissues has yet to be described. Here, we built constructs that express AtDRO1 fused with rapid-folding VENUS at either the N- or C-terminus, driven by a 2 kb fragment spanning the native *AtDRO1* promoter.

**Fig. 1:**
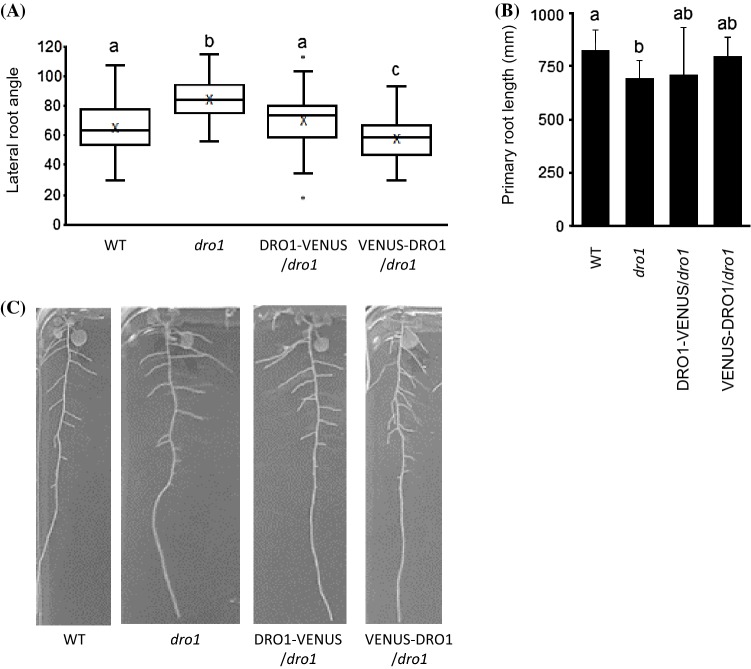
Expression of AtDRO1-VENUS or VENUS-AtDRO1 from the *AtDRO1* native promoter complements branch angle phenotype in *atdro1* mutants. **a** Box-plot distribution of branch angles quantified using Image J at 14-dpg. Different letters indicate a significant difference (Student's t-test p < 0.05). X indicates branch angle mean for each line. **b** Primary root length quantified using ImageJ at 14-dpg. Different letters indicate a significant difference (Student's *t* test p < 0.05). Bars ± SE. **c** Representative images of root architecture at 14-dpg

To determine the functionality of the AtDRO1-VENUS and VENUS-AtDRO1 constructs, we first assessed their ability to complement the *atdro1* root phenotype by identifying lines homozygous for both the VENUS-tagged transgene and the *atdro1* mutation and measuring lateral root branch angles. Consistent with previous results, *atdro1* mutants exhibited significantly wider branch angles and shorter primary root lengths than WT plants. Plants expressing AtDRO1-VENUS or VENUS-AtDRO1 from the *AtDRO1* native promoter both exhibited significantly narrower lateral roots angles than *atdro1* plants (Fig. 1). Furthermore, VENUS-AtDRO1 plants had significantly narrower lateral branch angles compared to AtDRO1-VENUS and WT plants. Consistent with previous results, *atdro1* primary roots were significantly shorter than WT roots. AtDRO1-VENUS and VENUS-AtDRO1 fully restored lateral root angles of the *atdro1* mutant and partially complemented root length as an intermediate primary root length was observed that was not significantly different from WT or *atdro1* roots. Together, this suggests AtDRO1-VENUS and VENUS-AtDRO1 when expressed from the *AtDRO1* native promoter can complement the *atdro1* phenotype.

### AtDRO1 localization

**Fig. 2:**
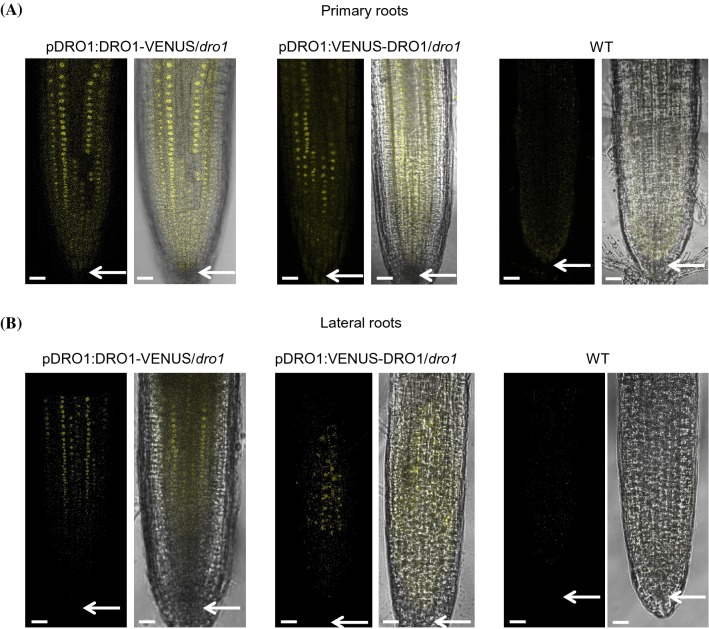
Localization of AtDRO1 in primary and lateral roots. **a** Primary roots from wild-type, pAtDRO1::AtDRO1-VENUS/*atdro1,* and pAtDRO1::VENUS-AtDRO1/*atdro1* seedlings imaged using confocal microscopy at 10-dpg. Scale bar, 20 µm. **b** Lateral roots from wild-type, pAtDRO1::AtDRO1-VENUS/*atdro1,* and pAtDRO1::VENUS-AtDRO1/*atdro1* seedlings imaged using confocal microscopy at 18-dpg. Scale bar, 20 µm. Arrows indicate region containing the columella

To determine the localization of AtDRO1 protein, roots of homozygous pAtDRO1::AtDRO1-VENUS/*atdro1* and pAtDRO1::VENUS-AtDRO1/*atdro1* were imaged using confocal microscopy. In biological replicates from two independent lines for each construct we observed a nuclear localization pattern with VENUS signal near the root tips in both primary and lateral roots (Fig. 2). Representative images are shown after observing multiple focal planes, and the localization pattern was the same whether VENUS was at the N- or C-terminus, although VENUS expression was brighter in pAtDRO1::AtDRO1-VENUS/*atdro1* lines. It is important to note that in some biological replicates no detectable VENUS signal was observed in primary or lateral roots. In roots with detectable signal, a relatively high detector gain was necessary to clearly distinguish the signal from background levels suggesting that AtDRO1 protein is not readily visualized possibly due to low levels and/or rapid protein turnover. Application of the proteasome chemical inhibitor MG132 to root tips did not enhance AtDRO1 visualization (data not shown). Nuclear expression was observed largely in cortical and endodermal tissue, and was unexpectedly undetectable in the columella, lateral root cap, and epidermal layers (Fig. 2, arrows indicate columella region).

### Gravity-induced auxin gradient formation in lateral roots is lost in *atdro1* single mutants

**Fig. 3:**
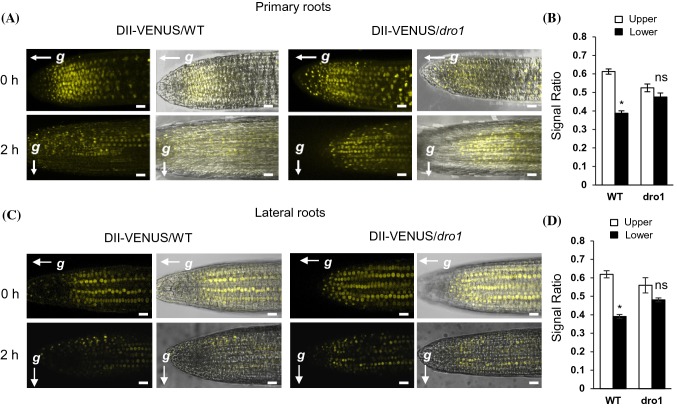
DII-VENUS localization in primary and lateral roots. **a** Primary wild-type and *atdro1* roots expressing DII-VENUS. Roots were imaged using confocal microscopy at 11-dpg before and after 2 h 90° reorientation. Scale bar, 20 µm. **b** Quantification of DII-VENUS fluorescent intensity in wild-type and *atdro1* primary roots. Fluorescent intensity was measured 2 h after 90° rotation. The fluorescent signal detected in the upper or lower half is shown relative to the fluorescent signal in the entire root tip. Bars represent means ± SE, n = 4 plants. The fluorescent intensity of DII-VENUS is inversely proportional to the auxin concentration. **c** Primary wild-type and *atdro1* roots expressing DII-VENUS. Roots were imaged using confocal microscopy at 18-dpg before and after 2 h 90° reorientation. Scale bar, 20 µm. **d** Quantification of DII-VENUS fluorescent intensity in wild-type and *atdro1* lateral roots. Fluorescent intensity was measured 2 h after 90° rotation. The fluorescent signal detected in the upper or lower half is shown relative to the fluorescent signal in the entire root tip. Bars represent means ± SE, n = 5 plants. Asterisks indicate Student's *t* test values of p < 0.05

It was previously reported that lateral auxin transport was partially reversed upon gravistimulation in the triple mutant *atdro1 atdro2 atdro3* (Taniguchi et al. 2017; Yoshihara and Spalding 2017; Ge and Chen 2019). To determine the role AtDRO1 specifically plays in this phenotype and to dissect the roles of multiple DRO1 proteins, we crossed *atdro1* mutants with DII-Venus, a sensor for auxin-induced protein degradation and inverse proxy for auxin distribution (Brunoud et al. 2012). WT or *atdro1* lines were examined by confocal microscopy before and 2 h after 90° rotation. As previously reported, WT plants established a new auxin gradient after gravistimulation with significantly less fluorescent signal in the lower half of the root (Fig. 3). In the *atdro1* mutant background the formation of a gradient was significantly impaired after reorientation, as there was no significant difference in fluorescent signal between the upper and lower half of the roots. This suggests loss of *AtDRO1* alone is enough to disrupt normal auxin gradient formation after gravistimulation but the loss of additional *DRO* genes is required for reversal of the auxin gradient.

### The auxin efflux protein PIN3 polarity was not altered in *atdro1* mutant under gravistimulation

**Fig. 4:**
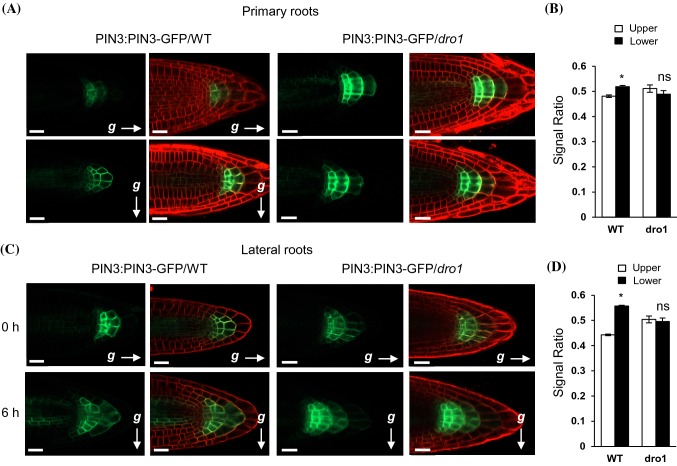
PIN3-GFP localization in primary and lateral roots. **a** Primary WT and *atdro1* roots expressing PIN3-GFP from the PIN3 native promoter counterstained with propidium iodide. Roots were imaged using confocal microscopy at 5-dpg before and after 6 h of 90° reorientation. Scale bar, 20 µm. **b** Quantification of PIN3-GFP fluorescent intensity in WT and *atdro1* primary roots measured after 6 h of 90° rotation. The fluorescent signal detected in the upper or lower half is shown relative to the fluorescent signal in the entire root tip. Bars represent means ± SE, n = 5–7 plants. **c** Lateral WT and *atdro1* roots expressing PIN3-GFP from the PIN3 native promoter counterstained with propidium iodide. Roots were imaged using confocal microscopy at 18-dpg before and after 6 h of 90° reorientation. Scale bar, 20 µm. **d** Quantification of PIN3-GFP fluorescent intensity in WT and *atdro1* lateral roots after 6 h of 90° rotation. The fluorescent signal detected in the upper or lower half is shown relative to the fluorescent signal in the entire root tip. Bars represent means ± SE, n = 4–7 plants. Asterisks indicate Student's *t* test values of p < 0.05

In response to gravistimulation, localization of the auxin efflux protein PIN3 polarizes to the lower side of columella cells and it’s domain expands towards the lower side of the root columella (Friml et al. 2002; Kleine-Vehn et al. 2010). In contrast, PIN3 polarity in *atdro1 atdro2 atdro3* triple mutant roots has been shown to expand towards the upper side of the root after gravistimulation (Taniguchi et al. 2017; Ge and Chen 2019). Following methods used by Taniguchi et al. (2017), to test whether PIN3 localization was altered in *atdro1* single mutant plants we crossed the reporter line pPIN3::PIN3-GFP with the *atdro1* mutant and selected for lines homozygous for the PIN3 reporter transgene and the *atdro1* mutation. The PIN3-GFP domain was then observed using confocal microscopy in WT and *atdro1* primary and lateral roots before or 6 h after 90° rotation (Fig. 4). As previously reported, WT plants show expansion of PIN3-GFP towards the lower side of the root. After gravistimulation GFP signal intensity was significantly greater in the lower half of WT primary and lateral roots. The PIN3-GFP domain in *atdro1* roots did not appear to change upon gravistimulation. There was no significant difference in GFP signal intensity between the lower and upper half of *atdro1* primary and lateral roots. Primary roots of *atdro1* mutants appear to show some upward expansion of the PIN3-GFP domain, however not to the same degree previously observed in triple mutants (Taniguchi et al. 2017).

### *atdro1* mutants exhibit a root auxin response and *AtDRO1* expression is not altered by exogenous auxin

**Fig. 5:**
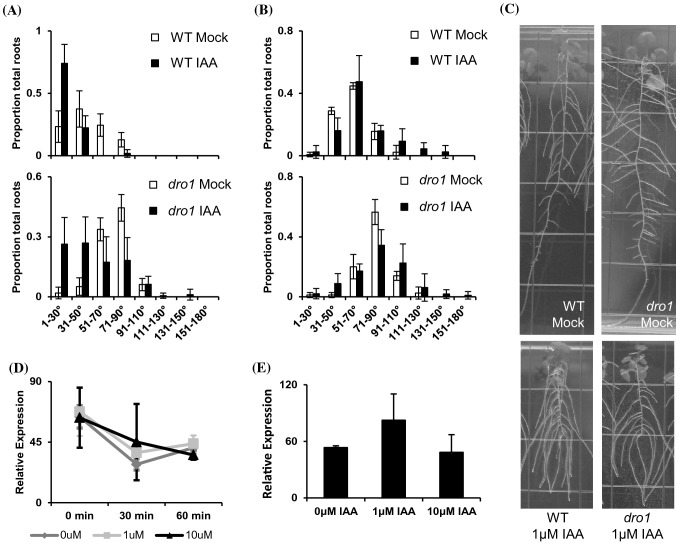
Auxin alters lateral root angles similarly in WT and *atdro1* mutants but does not affect *AtDRO1* expression. **a** WT and *atdro1* seedlings transplanted onto plates containing 1 μM IAA and allowed to continue growing for a week exhibited more root tips growing at a downward, narrow angle compared with controls. n = 4–6 plants. **b** WT and *atdro1* mutant seedlings grown on 1 μM IAA plates showed similar angles at root branch points compared to controls, but did have a broader distribution and more roots with wider branch angles than controls. n = 4–6 plants. **c** Representative images of root growth angles in plants treated with 1 μM IAA or mock. **d** Expression of *AtDRO1* measured in seedlings treated with mock, 1 μM or 10 μM IAA showed no significant difference at 0, 30, or 60 min after treatment. **e** Expression of *AtDRO1* in seedlings treated with mock, 1 μM IAA or 10 μM IAA showed no significant difference after 6 h of treatment

Previous research on *OsDRO1* in rice showed a decrease in gene expression in response to auxin treatment, which the authors correlated to binding of the OsARF1 transcriptional repressor to an Auxin Responsive Element (AuxRE) in the *OsDRO1* promoter (Uga et al. 2013). *AtDRO1* also has a full AuxRE upstream of the transcriptional start site, however at a greater distance than in *OsDRO1*. To better understand auxin-responsiveness, expression dynamics, and potential for auxin-related feedback loops involving *AtDRO1* in Arabidopsis, we treated seedlings with different concentrations of indole-3-acetic acid (IAA) and measured root angles and *AtDRO1* gene expression at different time points. It has been reported previously that growth on IAA results in more downward lateral root growth angles (Rosquete et al. 2013; Roychoudhry et al. 2017). Similar to these studies, we found that WT root tip angles were narrower when grown on 1uM IAA (Fig. 5a, c). When we measured the angle at the root branch points however, we found a broader distribution of roots angles, with more IAA-treated roots growing at wider angles (Fig. 5b, c). Though root angles were wider in *atdro1* mutant roots, we found that they responded in a similar manner to auxin treatment, with the population of tip angles in auxin-treated seedlings becoming narrower, and a similar broadening of the distribution of branch angles (Fig. 5a, b).

To measure gene expression in response to auxin, seedling roots were sprayed with IAA and collected after 30 min or 6 h. Although a decrease in expression was observed at 30 min in both the 1 μM and 10 μM treatments, this was not statistically different from the mock-treated control (Fig. 5d). A separate experiment measured *AtDRO1* expression 6 h after IAA treatment and found no significant differences between IAA treatments (Fig. 5e). These experiments demonstrate that *AtDRO1* shows little or no response to auxin treatment at the time points we assayed, and both WT and *atdro1* mutant plants show similar growth response to auxin.

### *atdro1* root tips have a number of auxin and root-related DEGs

To gain insight into *AtDRO1* influence on transcriptional networks we used an RNA-seq approach, comparing root tips of *atdro1* mutant seedlings to those of WT. As *AtDRO1* promoter expression is strongest near root tips of primary and middle to older lateral roots (Guseman et al. 2017) and because we observed protein localization in these same regions, we collected all root tips from populations of 10-day old seedlings that were grown on tissue culture plates. Five biological replicates, each containing all lateral root tips from eight seedlings, were used for RNA extractions from WT and *atdro1* plants (Fig. S1). Differentially Expressed Genes (DEGs) were identified after applying *t* tests across samples to identify genes that were consistently different between WT and *atdro1*, and subsequently filtered for > twofold change in expression. A total of 87 DEGs were identified (Table 1). Interestingly, *AtDRO1* was not differentially expressed, which we found was due to an upregulation of 5′ reads in the *atdro1* mutant, upstream of the T-DNA insertion, leading to relatively equal total numbers of reads between genotypes. Among the DEGs, we found several auxin-related genes as well as root development-related genes. These included *WOX11*, known to be involved in lateral root initiation and development and previously implicated in the rice LAZY1-mediated gravistimulation pathway (Zhang et al. 2018); *LRP1*, an auxin-responsive root development gene (Singh et al. 2019);and *WDL1*, which controls anisotropic cell expansion in roots (Yuen et al. 2003). A single striking DEG repressed over 2,000 fold in the *dro1* mutant was *MTO 1 RESPONDING DOWN* (*MRD1). MRD1* was previously identified as being downregulated in response to the overaccumulation of methionine in the *mto1-1* mutant (Goto and Naito 2002). *MRD1* overlaps with another gene *HEI10*, a RING/U-box ubiquitin ligase involved in recombination during meiosis (Chelysheva et al. 2012).

**Table 1:** Differentially expressed genes in *atdro1* mutant root tips as compared to WT

At ID	Gene name	Description	Fold change	*t* test
AT1G53480	MRD1	Encodes MRD1 (mto 1 responding down 1)	− 2713.59	1.44E−04
AT1G11720	SS3	Starch synthase 3	− 243.08	4.75E−02
AT5G14430	AT5G14430	S-adenosyl-l-methionine-dependent methyltransferases superfamily protein	− 213.21	4.38E−02
AT1G16340	KDSA2	Aldolase superfamily protein	− 151.42	2.89E−02
AT4G13100	AT4G13100	RING/U-box superfamily protein	− 130.46	4.76E−02
AT2G47470	UNE5	Thioredoxin family protein	− 105.69	1.87E−02
AT3G52050	AT3G52050	5′–3′ exonuclease family protein	− 78.69	4.76E−02
AT5G56850	AT5G56850	Unknown protein	− 58.71	1.65E−02
AT1G12240	BFRUCT4	Glycosyl hydrolases family 32 protein	− 43.85	1.38E−03
AT5G52570	BCH2	Beta-carotene hydroxylase 2	− 35.69	4.37E−02
AT1G65450	AT1G65450	HXXXD-type acyl-transferase family protein	− 20.49	3.27E−02
AT1G64710	AT1G64710	GroES-like zinc-binding dehydrogenase family protein	− 18.86	2.95E−02
AT5G52760	AT5G52760	Copper transport protein family	− 16.12	9.40E−04
AT4G02200	AT4G02200	Drought-responsive family protein	− 12.23	3.72E−02
AT4G01060	CPL3	CAPRICE-like MYB3	− 11.08	3.45E−02
AT4G27530	AT4G27530	Unknown protein	− 9.32	7.37E−03
AT4G05040	AT4G05040	Ankyrin repeat family protein	− 8.86	3.54E−04
AT1G02850	BGLU11	Beta glucosidase 11	− 8.7	2.26E−02
AT3G62650	AT3G62650	Unknown protein	− 8.46	3.23E−02
AT5G59810	SBT5.4	Subtilase family protein	− 7.94	4.02E−02
AT4G32080	AT4G32080	Unknown protein	− 6.98	4.69E−02
AT3G03660	WOX11	WUSCHEL related homeobox 11	− 6.51	6.26E−03
AT5G19390	AT5G19390	Rho GTPase activation protein (RhoGAP) with PH domain	− 5.79	5.66E−03
AT2G31810	AT2G31810	ACT domain-containing small subunit of acetolactate synthase protein	− 5.53	4.03E−02
AT2G33620	AT2G33620	AT hook motif DNA-binding family protein	− 5.07	4.23E−02
AT3G59430	AT3G59430	Unknown protein	− 4.85	2.25E−03
AT3G42725	AT3G42725	Putative membrane lipoprotein	− 4.73	4.18E−03
AT5G12330	LRP1	Encodes LRP1 (LATERAL ROOT PRIMORDIUM 1)	− 4.59	7.20E−03
AT3G06310	AT3G06310	Cox19-like CHCH family protein	− 4.32	2.60E−02
AT2G35660	CTF2A	FAD/NAD(P)-binding oxidoreductase family protein	− 4.29	3.45E−02
AT3G27300	G6PD5	Glucose-6-phosphate dehydrogenase 5	− 4.18	3.43E−02
AT2G23450	AT2G23450	Protein kinase superfamily protein	− 4.17	3.85E−02
AT3G03500	AT3G03500	TatD related DNase	− 4.15	1.18E−02
AT2G47830	AT2G47830	Cation efflux family protein	− 4	1.40E−02
AT1G55000	AT1G55000	Peptidoglycan-binding LysM domain-containing protein	− 3.94	3.60E−03
AT4G35920	MCA1	PLAC8 family protein	− 3.83	1.06E−02
AT3G27940	LBD26	LOB domain-containing protein 26	− 3.64	2.05E−04
AT1G63110	AT1G63110	GPI transamidase subunit PIG-U	− 3.55	8.12E−03
AT4G01450	AT4G01450	Nodulin MtN21/EamA-like transporter family protein	− 3.35	4.56E−02
AT1G73920	AT1G73920	Alpha/beta-Hydrolases superfamily protein	− 3.24	2.32E−02
AT2G16990	AT2G16990	Major facilitator superfamily protein	− 3.01	2.54E−02
AT4G27620	AT4G27620	Unknown protein	− 2.91	1.03E−02
AT5G08250	AT5G08250	Cytochrome P450 superfamily protein	− 2.86	4.57E−04
AT5G43500	ARP9	Actin-related protein 9	− 2.84	1.72E−02
AT5G40890	CLC-A	Chloride channel A	− 2.79	3.12E−02
AT1G52570	PLDALPHA2	Phospholipase D alpha 2	− 2.64	7.58E−03
AT1G80270	PPR596	PENTATRICOPEPTIDE REPEAT 596	− 2.64	2.44E−02
AT5G19430	AT5G19430	RING/U-box superfamily protein	− 2.54	3.77E−02
AT1G30450	CCC1	Cation-chloride co-transporter 1	− 2.53	2.94E−02
AT1G45688	AT1G45688	Unknown protein	− 2.52	1.94E−03
AT5G35940	AT5G35940	Mannose-binding lectin superfamily protein	− 2.31	1.32E−06
AT3G13030	AT3G13030	hAT transposon superfamily protein	− 2.31	1.70E−02
AT3G61490	AT3G61490	Pectin lyase-like superfamily protein	− 2.24	4.13E−02
AT1G53490	AT1G53490	RING/U-box superfamily protein	− 2.13	1.19E−04
AT3G54910	AT3G54910	RNI-like superfamily protein	2.43	3.92E−03
AT5G48010	THAS1	Thalianol synthase 1	2.45	1.25E−02
AT1G28210	ATJ1	DNAJ heat shock family protein	2.87	1.69E−02
AT5G09410	EICBP.B	Ethylene induced calmodulin binding protein	2.98	5.64E−03
AT5G01470	AT5G01470	S-adenosyl-l-methionine-dependent methyltransferases superfamily protein	3.07	3.58E−02
AT5G57700	AT5G57700	BNR/Asp-box repeat family protein	3.28	4.99E−02
AT2G18876	AT2G18876	Afadin/alpha-actinin-binding protein	3.41	3.52E−02
AT2G28930	PK1B	Protein kinase 1B	3.49	1.54E−02
AT4G03410	AT4G03410	Peroxisomal membrane 22 kDa (Mpv17/PMP22) family protein	4.03	2.68E−02
AT5G65080	MAF5	K-box region and MADS-box transcription factor family protein	4.12	1.18E−03
AT5G42410	AT5G42410	SAUR-like auxin-responsive protein family	4.61	3.08E−02
AT1G56220	AT1G56220	Dormancy/auxin associated family protein	4.61	4.67E−02
AT4G12720	NUDT7	MutT/nudix family protein	5.05	4.09E−04
AT4G24230	ACBP3	Acyl-CoA-binding domain 3	5.26	1.96E−03
AT5G06120	AT5G06120	ARM repeat superfamily protein	5.3	1.02E−02
AT2G43490	AT2G43490	Ypt/Rab-GAP domain of gyp1p superfamily protein	5.49	3.26E−02
AT3G04630	WDL1	WVD2-like 1	5.53	4.34E−02
AT3G46220	AT3G46220	Unknown protein	5.75	4.83E−02
AT1G29390	COR314-TM2	Cold regulated 314 thylakoid membrane 2	6.9	3.80E−02
AT1G23060	AT1G23060	Unknown protein	6.94	4.73E−04
AT1G33840	AT1G33840	Protein of unknown function (DUF567)	7.33	3.47E−02
AT4G34440	AT4G34440	Protein kinase superfamily protein	7.85	3.96E−02
AT1G07320	RPL4	Ribosomal protein L4	7.86	3.31E−02
AT4G01915	AT4G01915	Unknown protein	7.95	3.88E−03
AT1G52400	BGLU18	Beta glucosidase 18	14.83	3.55E−03
AT2G21230	AT2G21230	Basic-leucine zipper (bZIP) transcription factor family protein	16.56	1.45E−02
AT1G23860	RSZP21	RS-containing zinc finger protein 21	114.36	4.62E−02
AT4G13100	AT4G13100	RING/U-box superfamily protein	115.72	1.45E−02
AT5G04130	GYRB2	DNA GYRASE B2	127.33	2.70E−02
AT1G60460	AT1G60460	Unknown protein	129.52	1.98E−02
AT3G62620	AT3G62620	Sucrose-phosphate related	237.57	4.66E−02
AT2G22250	AAT	Aspartate aminotransferase	334.24	2.63E−02

RNA from pooled lateral and primary root tips, from populations of eight 14-day-old *atdro1* or WT seedlings, revealed a relatively small number DEGs. 87 DEGs fell under the criteria of showing 2 or more fold difference in expression after applying *t* tests among samples to find consistently differentially regulated transcripts. Five replicates of each genotype were used

## Discussion

*DRO* and *LAZY IGT* genes contribute to setting root and shoot growth trajectories in response to gravity, referred to as gravitropic set point angles (GSA), (Digby and Firn 1995). Here we show that the AtDRO1 protein is nuclear localized in roots tips when expressed from its native promoter. Our results contrast with prior studies that could not localize tagged AtDRO1 *in planta* when expressed under the native promoter. Using transient or inducible expression systems, DRO1 has repeatedly been found to be localized to the PM. In rice, Uga et al. (2013) showed that DRO1 was PM localized when transiently expressed in rice protoplasts, however, a naturally occurring truncated DRO1 derived from a shallow-rooting cultivar that lacked 25 amino acids spanning the conserved C-terminal domain V was localized to both the PM and the nucleus. Taniguchi et al. (2017) showed that a LZY3p:LZY3-mCherry construct (i.e. AtDRO1) could complement the *lazy1 lazy2 lazy3* triple mutant (i.e. *dro1, dro3, lazy1*) but they reported an inability to visualize the protein in root tips. More recently, Furutani et al. visualized this construct in cleared and fixed tissues, where it localized to the plasma membrane of columella cells, consistent with transient expression in Arabidopsis protoplasts, where the mCherry-tagged DRO1 protein was localized to the PM (Taniguchi et al. 2017; Furutani et al. 2020). However, in contrast to Uga et al. 2013, a truncated DRO1 lacking the conserved 14 amino-acid C-terminal domain (CCL) was still PM localized (Taniguchi et al. 2017). Ge and Chen (2016) reported that a NGR2-GFP (i.e. AtDRO1) construct driven by its native promoter could rescue a *ngr1 ngr2 ngr3* triple mutant (i.e. *dro1 dro2 dro3*) but they were unable to visualize protein localization in stably transformed Arabidopsis lines. Ge and Chen (2019) later reported that NGR2-GFP (ie. AtDRO1) was localized to the PM in tobacco leaf epidermal cells. These authors also showed PM localization when VENUS was inserted within a hydrophilic region of NGR2 and expressed in root cells under the control of an estradiol-inducible promoter (Ge and Chen 2019). The related IGT protein LAZY1 was found to be both PM and nuclear localized in shoots when expressed under a heat shock promoter, however, nuclear localization was not required for LAZY1 function (Yoshihara et al. 2013). In contrast, Li et al. (2019) demonstrated that rice LAZY1 lacking a nuclear localization signal was unable to rescue an *oslazy1* mutant phenotype. Sasaki and Yamamoto (2015) showed that AtLAZY1 is a peripheral PM protein but found that the C-terminal domain by itself associates with microtubules (Sasaki and Yamamoto 2015). Our observation that AtDRO1 protein was detectable in nuclei in root tips under native conditions suggests that localization to the PM is likely mediated by specific signals, cell-type specific interacting proteins, and/or conditions. A previous study with the maize LAZY1 protein (ZmLA1) found that it was capable of interacting with both IAA17 and a putative protein kinase, using yeast-2-hybrid and bimolecular fluorescence complementation (BiFC) (Dong et al. 2013). Interestingly, ZmLA1 interacted with IAA17 in the nucleus and with PKC at the plasma membrane. When the putative transmembrane domain (which also contains the conserved IGT motif) was deleted, ZmLA1 could no longer interact with PKC (Dong et al. 2013). It is possible that interactions with protein partners may also explain why DRO1 or LAZY1 have been found in both the nucleus and the PM in different assays. Both DRO and LAZY proteins contain a C-terminal EAR-like domain, which, when removed, abolishes ectopic overexpression phenotypes (Guseman et al. 2017). Recent work with DRO1 homologs in wheat used BiFC to show that this domain allows DRO1 to interact with TOPLESS at the PM and nucleus (Ashraf et al. 2019). More recently, work by Li et al. (2019) identified OsBRXL4 as a LAZY1 interactor that mediates OsLAZY1 plasma membrane localization. OsBRXL4 contains PH and FYVE domains predicted to mediate phospholipid interactions as well as RCC1 domains predicted to associate with chromatin. Their work led Li et al. to propose a model for rice LAZY1 whereby IGT proteins are shuttled between the nucleus and the PM via interactions with BRXL and/or other interacting proteins. Recently, similar BRXL proteins, referred to as RCC1-like domain (RLD) proteins, were shown to influence root branch angle in Arabidopsis, influence PIN protein localization, and polarly localize at the plasma membrane with PIN and LAZY or DRO proteins (Furutani et al. 2020). This work led to a model in which LAZY and DRO proteins control the localization of RLD proteins to the plasma membrane.

Work by Ge and Chen (2019) showed that *AtDRO1*, *AtDRO2*, and *AtDRO3* are expressed in the root cap and columella using stably transformed plant lines expressing nuclear localized GFP under the native *AtDRO* promoters. Similarly, Taniguchi et al. (2017) reported *AtDRO1* gene expression in the columella, as well as the stele above the elongation zone. This is consistent with our earlier work showing strong *pAtDRO1::GUS* expression in the columella and throughout the tips of primary roots as well as some middle and older aged lateral roots (Taniguchi et al. 2017; Yoshihara and Spalding 2017; Guseman et al. 2017). In young roots, *AtDRO1* expression was limited to the columella while no expression was found in newly emerging lateral roots (Guseman et al. 2017). We also noted exclusion of GUS expression in the columella of some older lateral roots, indicating a level of complexity in *AtDRO1* gene regulation. The importance of the complex expression patterns of *DRO1* was highlighted by Taniguchi et al. 2017 who showed that AtDRO1 driven by the *pSHR* or *pSCR* promoters, specific to the columella or stele, respectively, failed to rescue the *dro* triple mutant phenotype while a root-wide promoter, *pADF9* did rescue. The finding by Furutani et al. (2020), that fixed and cleared tissue exhibited DRO1 protein localization at the plasma membrane of columella cells, suggests that protein is found at low levels in these tissues, and may need to accumulate to be visualized, which may explain our inability to detect AtDRO1 protein in the regions of the root columella, lateral root cap, or epidermis. This could be due to degradation as part of its function resulting in the relative lack of detection. This could potentially occur through interaction with TOPLESS as reported by Ashraf et al. (2019). However, it is important to note we were unable to improve DRO1 visualization by addition of the proteasome inhibitor MG132. The nuclear localization we observed distal to the columella and lateral root cap may reveal additional properties or roles of AtDRO1, for example that the protein is mobile, either through transport or through interactions with a partner(s) and translocated more distally to a site of action distinct from the original site of expression. This has been shown to be the case for numerous transcription factors and other messenger RNAs moving through the phloem to their site of action (Hannapel et al. 2013; Long et al. 2015).

In contrast to the auxin-mediated downregulation of *OsDRO1* reported in rice, our experiments showed no strong change in *AtDRO1* gene expression in response to auxin (Fig. 5 and Uga et al. 2013). Multiple lines of reasoning may explain these differences. First, from phylogenetic sequence analyses, both Arabidopsis and rice contain multiple *DRO* genes, and the two genes in question may not be true orthologues. In fact, *AtDRO1* is closer to other rice *DRO* genes in a maximum likelihood analysis (Uga et al. 2013; Guseman et al. 2017). It may be the case that in Arabidopsis, *AtDRO2* and/or *AtDRO3* play a role as being auxin responsive. Second, Uga et al. (2013) identified one full TGTCTC Auxin Response Element (AuxRE), at position − 368 bp, and two core AuxREs, at positions − 86 and − 5 bp, in the promoter region of *OsDRO1*. These authors also demonstrated binding by OsARF1 to the region of the promoter containing the full AuxRE, which suggested this was the cause for *OsDRO1* repression in response to auxin. In Arabidopsis, we identified two full AuxREs, however their positions in the promoter of *AtDRO1* are at greater distance from the transcriptional start site, found at − 1950 bp and − 1287 bp (Guseman et al. 2017). This difference in distance may explain the contrasting auxin responsiveness between *OsDRO1* and *AtDRO1* expression. Finally, the two experiments used different types of auxins, 2,4-D in rice and IAA in Arabidopsis. It has been demonstrated that auxins have different properties (Delbarre et al. 1996; Tan et al. 2007; Calderón Villalobos et al. 2012), including affinities for auxin receptors and differences in the ability to be transported, which may also contribute to the contrasting results. The differences in auxin-responsiveness between rice and Arabidopsis DRO1 may further suggest the gain or loss of feedback loops between auxin signaling and the IGT gene family in different species.

Here, in single *atdro1* loss-of-function mutants, we observed impairment of DII-VENUS gradient establishment and of PIN3-GFP to exhibit downward domain expansion in response to gravistimulation, in both primary and lateral roots. Previous reports showed a reversal of the asymmetric distribution of both PIN3-GFP and DII-VENUS in triple *atlazy1 atdro1 atdro3* mutant roots in response to gravity. (Taniguchi et al. 2017; Yoshihara and Spalding 2017; Ge and Chen 2019). This loss of signal redistribution in the *atdro1* single mutant suggests that *AtDRO1* is required for establishing a polar auxin gradient in response to gravity and that *AtDRO2* and *AtDRO3* are not fully redundant with *AtDRO1* in this regard. Intriguingly, despite the lack of auxin redistribution, *atdro1* mutant plants do not exhibit gravitropic defects as measured via seedling primary root re-orientation experiments (Guseman et al., 2017). This may be due to only partial impairment of auxin gradient establishment, or may imply that there are differences between the gravitropic mechanisms that set GSA versus the root response to sudden re-orientation.

Our RNA-sequencing results in *atdro1* root tips identified a relatively small set of DEGs suggesting that broad transcriptional changes are not a primary mechanism for AtDRO1 action. However, 3 of the identified DEGs play known roles in anisotropic root elongation including WOX11 (− 6.5 fold), LRP1 (− 4.5 fold), and WDL1 (5.5 fold). Asymmetric induction of WOX11 by auxin in rice was shown to influence tiller angle downstream of LAZY1 and the double *wox6 wox11* mutant displayed impaired gravistimulation response (Zhang et al. 2018). WDL1 promotes right-handed helical root growth and was shown to influence slanting via anisotropic cell expansion associated with changes in cortical microtubules (Yuen et al. 2003). Auxin-mediated chromatin modification was shown to regulate the expression of LRP1 which acts downstream of ARFs to control root elongation and development (Singh et al. 2019). These DEGs highlight potential pathways that are disrupted by the loss of DRO1 function and represent future targets to better understand how root GSA is controlled.

## Materials and methods

### Plant material and growth conditions

Columbia (Col-0) was used as the WT line in all experiments. *atdro1* mutant seed was obtained from the Arabidopsis Biological Resource Center (https://abrc.osu.edu). One SALK line (SALK_201221C) and one SAIL line (SAIL_723_H11) were used. Both lines were genotyped as described previously (Guseman et al. 2017). The SALK insertion line was used for comparison in these experiments. DII-VENUS-N7 and PIN3::PIN3-GFP marker lines were provided by the Nemhauser lab. For phenotyping, seed were surface sterilized and sown on square plates containing half strength MS plates and 0.8% bactoagar. Plants were grown vertically to assay root architecture. Seeds were stratified on plates at 4 °C in darkness for 2 days, then transferred to growth chambers at 20 °C with a 16L/8D photoperiod.

### Plasmid construction and transgenic lines

A 2-kb fragment of the *AtDRO1* promoter, including the 5′ untranslated region (5′-UTR) was amplified from Arabidopsis genomic DNA and cloned into a modified pBINPLUS/ARS vector (Belknap et al. 2008) in place of the 35S promoter, using AscI and SalI restriction sites. The *AtDRO1* (At1g72490) coding region was amplified from Arabidopsis cDNA and VENUS was amplified from the DR5::VENUS-N7 vector. These were cloned downstream of the *AtDRO1* promoter with the VENUS fragment either N-terminal or C-terminal to the *AtDRO1* coding region, resulting in pAtDRO1::AtDRO1-VENUS and pAtDRO1::VENUS-AtDRO1. Primers were designed such that the fragment ends contained both restriction sites and proper sequence length and overlap to use for either restriction cloning or Gibson cloning. The N-terminal fragments contained an N-terminal SalI site and a C-terminal EcoRI site. C-terminal fragments contained an N-terminal XhoI site and a C-terminal BamHI site. Care was taken that the linker between both fragments excluded the stop codon and did not result in frameshifts. Constructs were transformed into an *atdro1* mutant background using the floral dip method (Clough and Bent 1998). Transformants were selected on half-strength MS agar plates containing Kanamycin. 10–12 T1 plants were selected per transformation and confirmed through genotyping. Representative T3 lines were used for analyses. For phenotypic analysis, T3 seeds were sown on square plates and grown vertically. Plates were imaged after 2 weeks and branch and tip angles were measured using ImageJ. Replicates were individual plants. DII-VENUS/*atdro1* and PIN3::PIN3-GFP/*atdro1* lines were generated by crossing and genotyping to obtain homozygous F3 lines used for this study.

### Microscopy

Confocal microscopy was performed on Zeiss LSM800 inverted laser scanning confocal microscope (Zeiss). For detection of improved YFP VENUS, (Nagai et al. 2002) an excitation wave length of 514 nm was used, and emission of 500 to 580 nm was used for detection. For propidium iodide excitation of 505 nm was used and emission of 585 to 700 nm. For PIN3-GFP excitation of 488 was used and emission of 450 to 560 nm. For VENUS experiments primary roots were imaged at 10 or 11-dpg. Lateral roots were imaged at 18-dpg. For PIN3-GFP experiments primary roots were taken at 5-dpg, lateral roots imaged at 18-dpg. For all experiments, seedlings imaged before reorientation were different from those imaged after reorientation. Fluorescent intensity of DII-VENUS and PIN3-GFP in WT and *atdro1* roots was measured using Image J (https://imagej.nih.gov/ij/).

### Hormone treatments

For growth angle experiments, WT and *atdro1* mutant seedlings were grown vertically for 7 days on MS plates, then were transplanted to plates containing 1 μM IAA or mock control (solvent, 95% ethanol). Seedlings were grown for 7 more days and then imaged. Branch and tip angles were taken with respect to the gravity vector and measured using ImageJ (https://imagej.nih.gov/ij/). Replicates were individual plants. N = 4–6 seedlings per treatment per genotype.

For expression studies, seedlings were sown and germinated on MS plates and grown vertically for 14 days. Seedling roots were then sprayed directly with an MS solution containing 1 μM IAA, 10 μM IAA or mock. After the indicated amount of time, roots from all seedlings from each plate were collected and flash frozen. Whole plates were used as replicates. N = 4 plates per experiment.

### Quantitative real-time PCR:

qPCR was performed as described previously (Guseman et al. 2017). Briefly, each reaction was run in triplicate using 50 ng of RNA in a 12-µl reaction volume, using the Super-script III Platinum SYBR Green qRT-PCR Kit (Invitrogen, now ThermoFisher Scientific, https://www.thermofisher.com). The reactions were performed using a 7900 DNA sequence detector (Applied Biosystems, now ThermoFisher Scientific, https://www.thermofisher.com). Quantification for Arabidopsis samples was performed using a standard curve derived from a serially diluted WT control RNA run in parallel.

### RNA-sequencing and analysis

Root tips were collected from *atdro1* SALK mutant (SALK_201221C) and WT 10 day-old seedlings. For each replicate, 2–3 mm of all root tips (primary and lateral) were removed, using a razor blade, from 6 individual seedlings and pooled. RNA was then extracted from five replicates of each genotype, using the DirectZol RNA Extraction Kit (Zymo Research, https://www.zymoresearch.com). Samples were analyzed and sequenced by MOgene to obtain 75 bp paired-end reads (https://www.mogene.com).

A total of 153,006,802 reads were obtained for the 5 WT samples and 155,267,246 reads were obtained for the 5 *atdro1* samples. For analysis, the RNA sequencing and transcriptomics analysis tools within the CLC Genomics Workbench ver 20.0 was used with default settings and the TAIR 10 genome as reference. P-value and *t* test cutoffs of 0.05 were applied and the remaining DEGs were filtered for those with > 2-fold change in expression (Qiagen, Germantown, MD).

## Electronic supplementary material

Below is the link to the electronic supplementary material.

Supplementary file1 (PDF 1387 kb)

## References

[CR1] Ashraf A, Rehman OU, Muzammil S, Léon J, Naz AA, Rasool F, Ali GM, Zafar Y, Khan MR (2019). Evolution of deeper rooting 1-like homoeologs in wheat entails the C-terminus mutations as well as gain and loss of auxin response elements. PLoS ONE.

[CR2] Belknap WR, Rockhold DR, McCue KF (2008). pBINPLUS/ARS: an improved plant transformation vector based on pBINPLUS. Biotechniques.

[CR3] Brunoud G, Wells DM, Oliva M, Larrieu A, Mirabet V, Burrow AH, Beeckman T, Kepinski S, Traas J, Bennett MJ, Vernoux T (2012). A novel sensor to map auxin response and distribution at high spatio-temporal resolution. Nature.

[CR4] Calderón Villalobos LIA, Lee S, De Oliveira C, Ivetac A, Brandt W, Armitage L, Sheard LB, Tan X, Parry G, Mao H, Zheng N, Napier R, Kepinski S, Estelle M (2012). A combinatorial TIR1/AFB-Aux/IAA co-receptor system for differential sensing of auxin. Nat Chem Biol.

[CR5] Chelysheva L, Vezon D, Chambon A, Gendrot G, Pereira L, Lemhemdi A, Vrielynck N, Le Guin S, Novatchkova M, Grelon M (2012). The arabidopsis HEI10 is a new ZMM protein related to Zip3. PLoS Genet.

[CR6] Clough SJ, Bent AF (1998). Floral dip: a simplified method for agrobacterium-mediated transformation of *Arabidopsis thaliana*. Plant J.

[CR7] Dardick C, Callahan A, Horn R, Ruiz KB, Zhebentyayeva T, Hollender C, Whitaker M, Abbott A, Scorza R (2013). PpeTAC1 promotes the horizontal growth of branches in peach trees and is a member of a functionally conserved gene family found in diverse plants species. Plant J.

[CR8] Delbarre A, Muller P, Imhoff V, Guern J (1996). Comparison of mechanisms controlling uptake and accumulation of 2,4-dichlorophenoxy acetic acid, naphthalene-1-acetic acid, and indole-3-acetic acid in suspension-cultured tobacco cells. Planta.

[CR9] Digby J, Firn RD (1995). The gravitropic set-point angle (GSA): the identification of an important developmentally controlled variable governing plant architecture. Plant Cell Environ.

[CR10] Dong Z, Jiang C, Chen X, Zhang T, Ding L, Song W, Luo H, Lai J, Chen H, Liu R, Zhang X, Jin W (2013). Maize LAZY1 mediates shoot gravitropism and inflorescence development through regulating auxin transport, auxin signaling, and light response. Plant Physiol.

[CR11] Friml J, Wiśniewska J, Benková E, Mendgen K, Palme K (2002). Lateral relocation of auxin efflux regulator PIN3 mediates tropism in Arabidopsis. Nature.

[CR12] Furutani M, Hirano Y, Nishimura T, Nakamura M, Taniguchi M, Suzuki K, Oshida R, Kondo C, Sun S, Kato K, Fukao Y, Hakoshima T, Morita MT (2020). Polar recruitment of RLD by LAZY1-like protein during gravity signaling in root branch angle control. Nat Commun.

[CR13] Ge L, Chen R (2016). Negative gravitropism in plant roots. Nat Plants.

[CR14] Ge L, Chen R (2019). Negative gravitropic response of roots directs auxin flow to control root gravitropism. Plant Cell Environ.

[CR15] Goto DB, Naito S (2002). AtMRD1 and AtMRU1, two novel genes with altered mRNA levels in the methionine over-accumulating mto1-1 mutant of *Arabidopsis thaliana*. Plant Cell Physiol.

[CR16] Guseman JM, Webb K, Srinivasan C, Dardick C (2017). *DRO1* influences root system architecture in Arabidopsis and Prunus species. Plant J.

[CR17] Hannapel DJ, Sharma P, Lin T (2013). Phloem-mobile messenger RNAs and root development. Front Plant Sci.

[CR18] Hollender CA, Dardick C (2015). Molecular basis of angiosperm tree architecture. New Phytol.

[CR19] Kagale S, Rozwadowski K (2010). Small yet effective: the ethylene-responsive element binding factor-associated amphiphilic repression (EAR) motif. Plant Signal Behav.

[CR20] Kleine-Vehn J, Ding Z, Jones AR, Tasaka M, Morita MT, Friml J (2010). Gravity-induced PIN transcytosis for polarization of auxin fluxes in gravity-sensing root cells. Proc Natl Acad Sci U S A.

[CR21] Kramer PJ (1983). Water relations of plants.

[CR22] Li P, Wang Y, Qian Q, Fu Z, Wang M, Zeng D, Li B, Wang X, Li J (2007). LAZY1 controls rice shoot gravitropism through regulating polar auxin transport. Cell Res.

[CR23] Li Z, Liang Y, Yuan Y, Wang L, Meng X, Xiong G, Zhou J, Cai Y, Han N, Hua L, Liu G, Li J, Wang Y (2019). OsBRXL4 regulates shoot gravitropism and rice tiller angle through affecting LAZY1 nuclear localization. Mol Plant.

[CR24] Long Y, Scheres B, Blilou I (2015). The logic of communication: roles for mobile transcription factors in plants. J Exp Bot.

[CR25] Lynch JP (2013). Steep, cheap and deep: an ideotype to optimize water and N acquisition by maize root systems. Ann Bot.

[CR26] Nagai T, Ibata K, Park ES, Kubota M, Mikoshiba K, Miyawaki A (2002). A variant of yellow fluorescent protein with fast and efficient maturation for cell-biological applications. Nat Biotechnol.

[CR27] Rosquete MR, Von Wangenheim D, Marhavý P, Barbez E, Stelzer EHK, Benková E, Maizel A, Kleine-Vehn J (2013). An auxin transport mechanism restricts positive orthogravitropism in lateral roots. Curr Biol.

[CR28] Roychoudhry S, Kepinski S (2015). Shoot and root branch growth angle control-the wonderfulness of lateralness. Curr Opin Plant Biol.

[CR29] Roychoudhry S, Kieffer M, Del Bianco M, Liao CY, Weijers D, Kepinski S (2017). The developmental and environmental regulation of gravitropic setpoint angle in Arabidopsis and bean. Sci Rep.

[CR30] Sasaki S, Yamamoto KT (2015). Arabidopsis LAZY1 is a peripheral membrane protein of which the carboxy-terminal fragment potentially interacts with microtubules. Plant Biotechnol.

[CR31] Singh S, Yadav S, Singh A, Mahima M, Singh A, Gautam V, Sarkar AK (2019). Auxin signaling modulates LATERAL ROOT PRIMORDIUM1 (LRP1) expression during lateral root development in Arabidopsis. Plant J.

[CR32] Tan X, Calderon-Villalobos LIA, Sharon M, Zheng C, Robinson CV, Estelle M, Zheng N (2007). Mechanism of auxin perception by the TIR1 ubiquitin ligase. Nature.

[CR33] Taniguchi M, Furutani M, Nishimura T, Nakamura M, Fushita T, Iijima K, Baba K, Tanaka H, Toyota M, Tasaka M, Morita MT (2017). The arabidopsis LAZY1 family plays a key role in gravity signaling within statocytes and in branch angle control of roots and shoots. Plant Cell.

[CR34] Uga Y, Sugimoto K, Ogawa S, Rane J, Ishitani M, Hara N, Kitomi Y, Inukai Y, Ono K, Kanno N, Inoue H, Takehisa H, Motoyama R, Nagamura Y, Wu J, Matsumoto T, Takai T, Okuno K, Yano M (2013). Control of root system architecture by DEEPER ROOTING 1 increases rice yield under drought conditions. Nat Genet.

[CR35] Yoshihara T, Iino M (2007). Identification of the gravitropism-related rice gene LAZY1 and elucidation of LAZY1-dependent and -independent gravity signaling pathways. Plant Cell Physiol.

[CR36] Yoshihara T, Spalding EP (2017). LAZY genes mediate the effects of gravity on auxin gradients and plant architecture. Plant Physiol.

[CR37] Yoshihara T, Spalding EP (2019). Switching the direction of stem gravitropism by altering two amino acids in AtLAZY1. Plant Physiol.

[CR38] Yoshihara T, Spalding EP, Iino M (2013). AtLAZY1 is a signaling component required for gravitropism of the *Arabidopsis thaliana* inflorescence. Plant J.

[CR39] Yuen CYL, Pearlman RS, Silo-Suh L, Hilson P, Carroll KL, Masson PH (2003). WVD2 and WDL1 modulate helical organ growth and anisotropic cell expansion in Arabidopsis1[w]. Plant Physiol.

[CR40] Zhang N, Yu H, Yu H, Cai Y, Huang L, Xu C, Xiong G, Meng X, Wang J, Chen H, Liu G, Jing Y, Yuan Y, Liang Y, Li S, Smith SM, Li J, Wang Y (2018). A core regulatory pathway controlling rice tiller angle mediated by the LAZY1-dependent asymmetric distribution of auxin. Plant Cell.

