# Supplementary material for: AtDRO1 is nuclear localized in root tips under native conditions and impacts auxin localization
Source: Plant Mol Biol. 2020 Mar 4;103(1):197–210. doi: 10.1007/s11103-020-00984-2 (PMC7170825; doi:10.1007/s11103-020-00984-2)
Supplement: Supplementary file 1 — Supplementary file1 (PDF 1387 kb) [file 11103_2020_984_MOESM1_ESM.pdf]

SUPPLEMENTAL MATERIAL

AtDRO1 is nuclear localized in root tips under native conditions and impacts auxin localization

Jessica M. Waite[1,2#], Tamara D. Collum[2#], and Chris Dardick*[2]

[1] Washington State University Tree Fruit Research and Extension Center, Wenatchee, WA 98801, USA [2]USDA-ARS Appalachian Fruit Research Station, Kearneysville, WV 25430, USA.

[#]authors equaling contributed to this work

*corresponding author

chris.dardick@usda.gov

Supplemental Table 1: IGT family gene IDs and naming conventions.

**AT ID →**	**At1g72490**	**At1g19115**	**At1g17400**	**At5g14090**	**At3g27025**	**At2g46640**	**At3g24750**
Current Study	DRO1	DRO2	DRO3	LAZY1	LAZY6	TAC1	
Uga et al. 2013	Closest A. thaliana homolog to OsDRO1						
Yoshihara et al. 2013	LAZY1 homolog	LAZY1 homolog	LAZY1 homolog	LAZY1	LAZY1 homolog		LAZY1 homolog
Dardick et al. 2013				LAZY1		TAC1	
Ge and Chen 2016	NGR2	NGR3	NGR1				
Guseman et al. 2017	DRO1	DRO2	DRO3				
Yoshihara and Spalding 2017	LAZY4	LAZY3	LAZY2	LAZY1	LAZY6		LAZY5
Taniguchi et al. 2017	LZY3		LZY2	LZY1			

SUPPLEMENTARY FIGURE LEGENDS

Supplementary Figure S1.

Representative Arabidopsis seedlings before and after removing lateral root tips for RNA sequencing. The most distal 2-3 mm of all lateral roots were removed from WT and *atdro1* seedlings and collected for RNA extraction and sequencing. Intact WT seedlings are shown on the left, and on the right the same seedlings with lateral root tips removed.

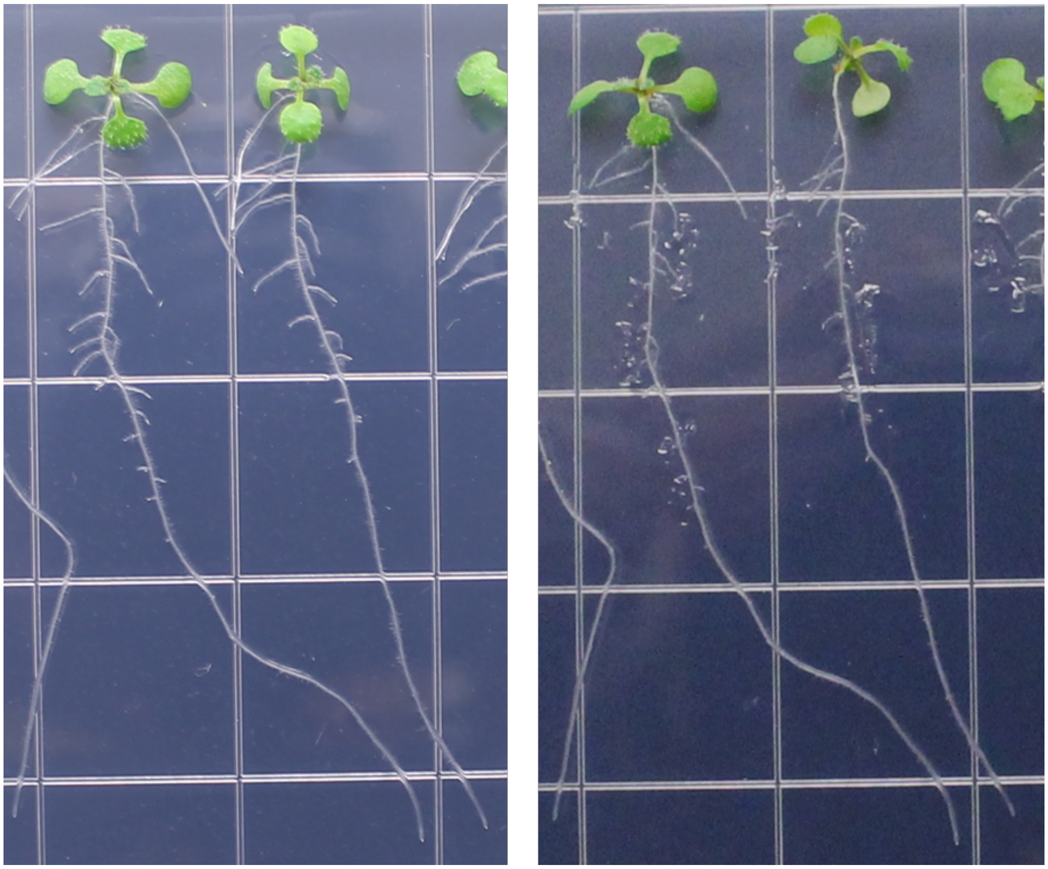

Supplementary Figure S1
